# Evaluating the Role of MC1R Variants in Cutaneous and Ocular Phenotypes: A Candidate TYR–MC1R Genetic Interaction in Oculocutaneous Disorders

**DOI:** 10.3390/genes17101289

**Published:** 2026-10-15

**Authors:** Enzo Di Iorio, Francesco Parmeggiani, Antonella De Nardi, Francesco Prevedello, Rebecca Affuso, Anna Demarinis, Andrea Gazzin, Chiara Rigon, Vanessa Barbaro, Katia De Nadai, Antonio Cartabellotta, Ginevra Giovanna Adamo, Marco Mura, Agnese Suppiej, Giorgio Palù, Leonardo Salviati, Marco Tavolato

**Affiliations:** 1 Department of Molecular Medicine, University of Padova, 35121 Padova, Italy; anna.demarinis@unipd.it (A.D.); giorgio.palu@unipd.it (G.P.); 2 Clinical Genetics Unit, Department of Integrated Diagnostic Services, University Hospital of Padova, 35128 Padova, Italy; andrea.gazzin@aopd.veneto.it (A.G.); chiara.rigon@unipd.it (C.R.); leonardo.salviati@unipd.it (L.S.); 3 Center for Retinitis Pigmentosa of Veneto Region (ERN-EYE Network), Camposampiero Hospital, Azienda ULSS 6 Euganea, 35121 Padova, Italy; prmfnc@unife.it (F.P.); katia.denadai@unife.it (K.D.N.); agnese.suppiej@unife.it (A.S.); marco.tavolato@aulss6.veneto.it (M.T.); 4 Department of Translational Medicine and for Romagna, University of Ferrara, 44121 Ferrara, Italy; antonio.cartabellotta@unife.it (A.C.); ginevragiovanna.adamo@unife.it (G.G.A.); marco.mura@unife.it (M.M.); 5 Medical Genetics Unit, Treviso Hospital, 31100 Treviso, Italy; antonella.denardi@aulss2.veneto.it; 6 Department of Women’s and Children’s Health, University of Padova, 35121 Padova, Italy; francesco.prevedello@aopd.veneto.it (F.P.); rebecca.affuso@aopd.veneto.it (R.A.); 7 Fondazione Banca degli Occhi del Veneto ETS, 30174 Venice, Italy; vanessa.barbaro@fbov.it; 8 King Khaled Eye Specialist Hospital, Riyadh 12211, Saudi Arabia; 9 Department of Medical Sciences, University of Ferrara, 44121 Ferrara, Italy; 10 Robert Hollman Foundation, 35121 Padova, Italy; 11 Fondazione Banca dei Tessuti del Veneto ETS, 31100 Treviso, Italy

**Keywords:** *MC1R* gene, *TYR* gene, digenic inheritance, skin tumors, foveal hypoplasia, ocular albinism

## Abstract

**Background:** The melanocortin-1 receptor (*MC1R*) gene is a major determinant of skin and hair color. Although several *MC1R* variants increase the risk of skin tumors regardless of pigmentation, their role as genetic modifiers in ocular diseases remains poorly understood. **Methods:** Here, we analyzed a cohort of 48 Caucasian individuals using a custom next-generation sequencing (NGS) panel targeted for oculocutaneous disorders. **Results:** *MC1R* variants were identified in two probands selected from this cohort, exhibiting distinctly divergent pathological phenotypes. The first patient, an adult male with a pheomelanin-related trait and a history of early-onset skin cancer without ocular abnormalities, carried compound heterozygous *MC1R* variants: the novel frameshift c.537dupC (p.Ile180Hisfs*59) and the known c.880G>C (p.Asp294His). Conversely, the second patient, a young boy with severe vision-threatening features—including congenital nystagmus, foveal hypoplasia, and an atypical visual-pathway misrouting pattern—carried a candidate digenic genotype combining the same *MC1R* c.880G>C variant with the albinism-related c.1209G>T (p.Arg403Ser) variant in the tyrosinase (*TYR*) gene. **Conclusions:** While *MC1R* variants have previously been proposed as phenotypic modifiers in albinism, primarily in combination with the oculocutaneous type II melanosomal transmembrane protein (*OCA2*) encoding gene, our findings support a potential role for *MC1R* as a candidate modifier interacting with *TYR* in neuroretinal development. Although functional validation is required to establish causality, incorporating *MC1R* screening in patients with unresolved foveal hypoplasia may help clarify complex oligogenic mechanisms and optimize phenotypic classification.

## 1. Introduction

The melanocortin-1 receptor (*MC1R*) gene (16q24.3, OMIM #155555) encodes a G protein-coupled receptor that plays a pivotal role in regulating melanin synthesis. Upon activation by ultraviolet (UV) light and subsequent alpha-melanocyte-stimulating hormone (α-MSH) signaling, MC1R promotes the production of photoprotective eumelanin. Conversely, loss-of-function variants disrupt this pathway, favoring the synthesis of pro-oxidative pheomelanin [1,2]. Consequently, MC1R acts as a major determinant of human pigmentation diversity, driving variations in hair color, skin tone, and UV sensitivity [3,4]. Although highly polymorphic, specific high-penetrance variants—such as p.Arg151Cys, p.Arg160Trp, and p.Asp294His—exhibit population-specific distributions and are strongly associated with the red hair phenotype [5]. The shift toward pheomelanogenesis compromises the cellular shielding capacity against UV radiation, thereby accelerating the accumulation of intracellular reactive oxygen species (ROS). Accordingly, *MC1R* variants have been shown to significantly increase the risk of melanoma skin cancer (MSC) in both dark- and light-pigmented individuals. Notably, the risk conferred to carriers compared to non-carriers suggests that these variants may partially mediate oncogenesis through pathways independent of macroscopic pigmentation and UV exposure [6,7,8,9,10]. Furthermore, MC1R is known to interact epistatically with major melanoma-susceptibility genes, including *CDKN2A* and *BRAF* [11]. Regarding non-melanoma skin cancer (NMSC), carriers of two *MC1R* variant alleles exhibit a 2- to 3-fold increased risk of basal cell and squamous cell carcinomas, though the precise contribution of individual variants to specific tumor histotypes remains to be fully elucidated [11,12,13]. Moreover, *MC1R* variants are highly prevalent in non-albino Caucasian populations, where they modulate lighter pigmentation phenotypes even in the heterozygous state [14]. Given its cooperative role within the melanogenic cascade, *MC1R* has also been investigated as a genetic modifier in hypopigmentation disorders. Specifically, its variants have been shown to interact concomitantly with mutations in the oculocutaneous albinism type II melanosomal transmembrane protein (*OCA2*) gene to influence human pigmentary outcomes [15,16,17,18]. Alongside *MC1R* and *OCA2*, the tyrosinase (*TYR*) gene stands out as a core determinant of skin color and represents one of the most frequently mutated causative genes in albinism. Albinism encompasses a heterogeneous group of at least 22 clinical conditions (categorized into syndromic, non-syndromic, and isolated ocular forms) arising from mutations across 23 distinct genes. These conditions are universally characterized by a congenital reduction or total absence of melanin pigment in the skin, hair, and eyes, with a clinical severity that strictly depends on the specific gene affected and the nature of the mutational hit [19,20,21,22,23]. Non-syndromic albinism is broadly classified into two main clinical entities, including X-linked forms: oculocutaneous albinism (OCA) and ocular albinism (OA). Both display extensive genotypic and phenotypic heterogeneity. While OCA typically allows for a straightforward clinical diagnosis due to unequivocal cutaneous and hair hypopigmentation, OA can be far subtler. This is particularly true in pediatric patients who lack a severe or complete albino phenotype. In these individuals, the distinct ocular alterations may manifest with very mild phenotypic expressivity [24,25,26,27,28], often leaving the chiasmal misrouting of optic nerve fibers—detectable using visual evoked potentials (VEPs)—as the sole pathognomonic sign of the disease [29,30,31,32,33,34]. To date, the mutational spectrum of the *MC1R* gene has been only occasionally investigated in patients presenting with albino or albino-like phenotypes [35,36,37,38,39,40]. In a large OCA/OA cohort, Preising and collaborators reported the co-occurrence of *MC1R* variants alongside heterozygous *OCA2* mutations, inferring a potentially altered *OCA2*–*MC1R* molecular interaction driven by the MC1R p.Val60Leu mutant [35]. However, the potential cooperative role of *MC1R* variants in patients carrying causative mutations in the *TYR* gene, and its subsequent impact on severe ocular development, has not yet been documented. In this investigational context, we describe novel monogenic and digenic genotype–phenotype correlations that reveal the pathological role of *MC1R* variants in two distinct patients. Our findings highlight how *MC1R* variants can act either within a monogenic framework associated with increased skin-cancer susceptibility, or as a candidate phenotypic modifier potentially contributing to complex ocular phenotypes.

## 2. Results

### Screening Background and Case Identification

To evaluate specific genotype–phenotype correlations in oculocutaneous disorders, a screening background comprising 48 Caucasian individuals was previously analyzed using a custom Next-Generation Sequencing (NGS) panel targeting loci associated with hypopigmentation and albinism. While the overall clinical and statistical characteristics of this full cohort fall outside the scope of the present study, this screening served as a descriptive background to identify two unrelated probands carrying notable *MC1R* mutational patterns. Because these two individuals were specifically selected for their unusual multigenic profiles, these cases represent detailed genotype–phenotype observations rather than an estimate of prevalence or diagnostic yield. These two specific cases are detailed below to illustrate their distinct clinical phenotypes, representing monogenic and digenic inheritance models, respectively.

**Case 1: Novel Monogenic Compound Heterozygosity in *MC1R* and Cutaneous Phenotype with History of Skin Cancer**

**Figure 1:**
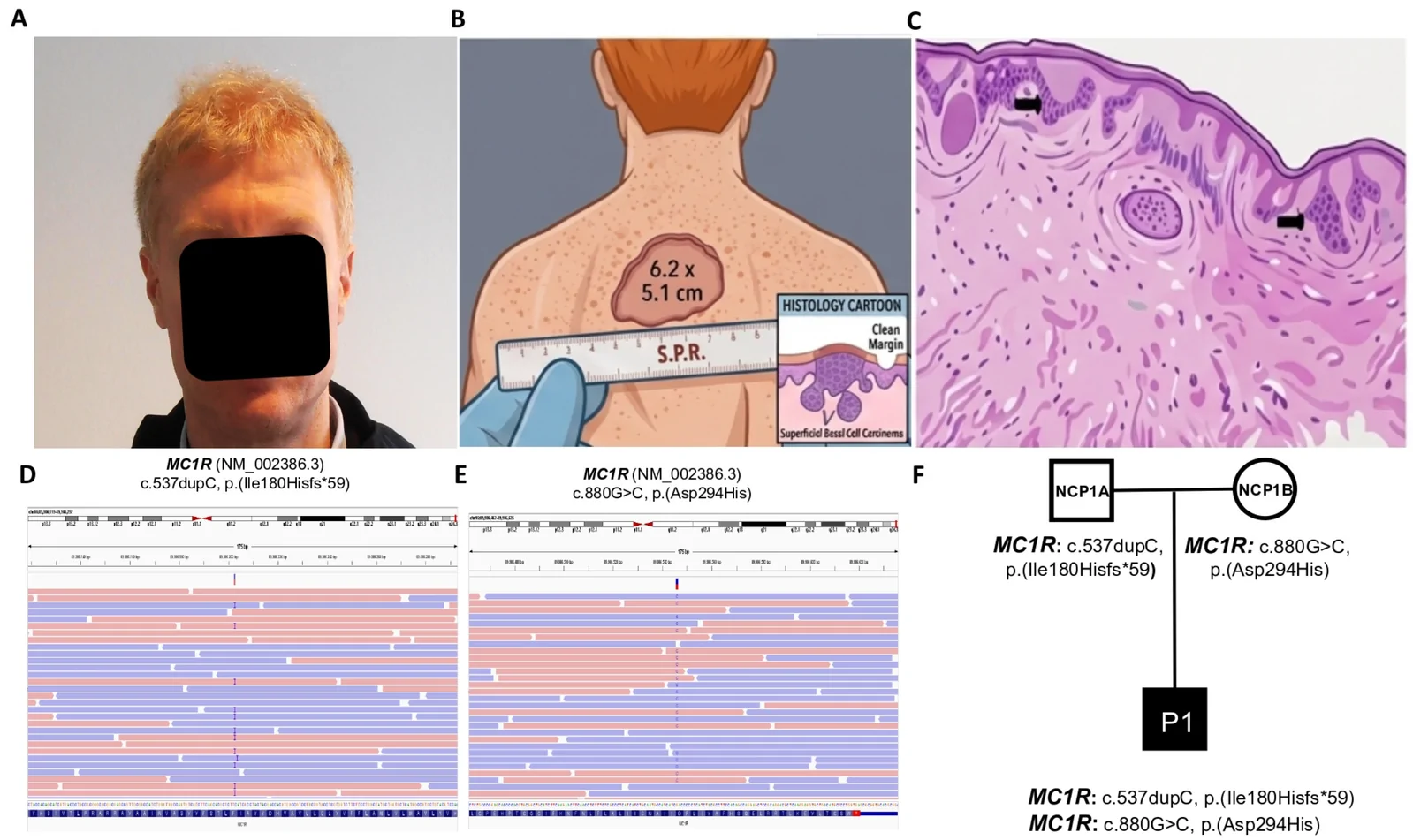
Clinical, genetic, and histopathological characterization of Proband P1. (**A**) Phenotypic appearance of the 50-year-old male proband (P1) displaying classic pheomelanin-related traits, including fair skin with ephelides (freckles), bright red hair, and light-colored irises, in the absence of ocular pathognomonic signs of albinism. (**B**) Schematic illustration representing the macroscopic presentation of the giant basal cell carcinoma (GBCC) on the patient’s upper back/shoulder area, measuring 6.2 × 5.1 centimeters prior to surgical excision at age 30. (**C**) Histopathological diagrammatic representation of the cutaneous lesion, highlighting the classic architectural features of basal cell carcinoma, including nests of atypical basaloid cells extending from the epidermis into the dermis (black arrows) accompanied by melanin pigmentation clusters. (**D**,**E**) Next-generation sequencing (NGS) visualization (Integrative Genomics Viewer, IGV) of target regions within the *MC1R* gene, demonstrating a monogenic compound heterozygosity: (**D**) the known missense variant c.880G>C (p.Asp294His) and (**E**) the novel frameshift insertion c.537dupC (p.Ile180Hisfs*59)—red arrows indicate the specific genomic coordinates of the variants. (**F**) Restricted family pedigree and data from trio segregation analysis confirming compound heterozygosity with variants inherited in trans: the paternally inherited *MC1R*-c.537dupC (NCP1A) and the maternally inherited *MC1R*-c.880G>C (NCP1B). Abbreviations: *MC1R*: Melanocortin 1 receptor.

A 50-year-old man (proband P1) was referred for genetic counseling and custom multigene panel analysis to investigate pigmentary abnormalities (Figure 1). Proband P1 exhibited classic pheomelanin-related traits, including bright red hair, fair skin with freckles, and blue-green irises, in the absence of pathognomonic clinical signs of either OCA or OA (Figure 1A; Table 1). Anamnestic medical history retrieval revealed that the patient had previously developed a juvenile giant basal cell carcinoma (GBCC), which had been successfully treated via surgical excision at 30 years of age, resulting in a recurrence-free follow-up lasting 20 years (Figure 1B,C). His ophthalmological history was unremarkable, consisting exclusively of excimer laser corneal refractive surgery for the correction of moderate myopia in both eyes, with no further ocular abnormalities. Targeted genetic screening using our custom NGS panel for pigmentary disorders identified monogenic compound heterozygous variants in the *MC1R* gene: the novel frameshift variant c.537dupC (p.Ile180Hisfs*59) and the known missense variant c.880G>C (p.Asp294His), extensively documented for its ability to increase susceptibility to non-melanoma skin cancers (NMSC) [11,12,13], one of the most dangerous forms of which is the GBCC, as observed in our patient (Figure 1D,E). This variant described for the first time was absent from general population databases and was classified as likely pathogenic according to the guidelines of the American College of Medical Genetics and Genomics (ACMG) [41]. No further pathogenic variants were detected in *TYR*, *OCA2*, or any other albinism-related genes included in the panel. While *MC1R* variants are frequently found in oncological cohorts presenting specific yellow/red pigmentary traits [1,2,3,4,5,6,7,8,9,10], it has never been previously reported in a case of GBCC, a rare and aggressive type of NMSC characterized by a diameter greater than or equal to 50 millimeters [42]. Family-based trio segregation analysis confirmed that P1 carries compound heterozygous *MC1R* variants inherited in trans (Figure 1F), with the *MC1R* c.537dupC variant being paternally inherited (NCP1A) and the *MC1R* c.880G>C variant being maternally inherited (NCP1B). Although both non-consanguineous parents of P1 presented darker phototypes compared to their son, both reported a positive personal history of late-onset melanoma skin cancer (MSC) (Table 1) [6,7,8].

**Case 2: Novel Digenic TYR / MC1R Inheritance and Ocular Phenotype with Foveal Hypoplasia and Congenital Nystagmus**

**Figure 2:**
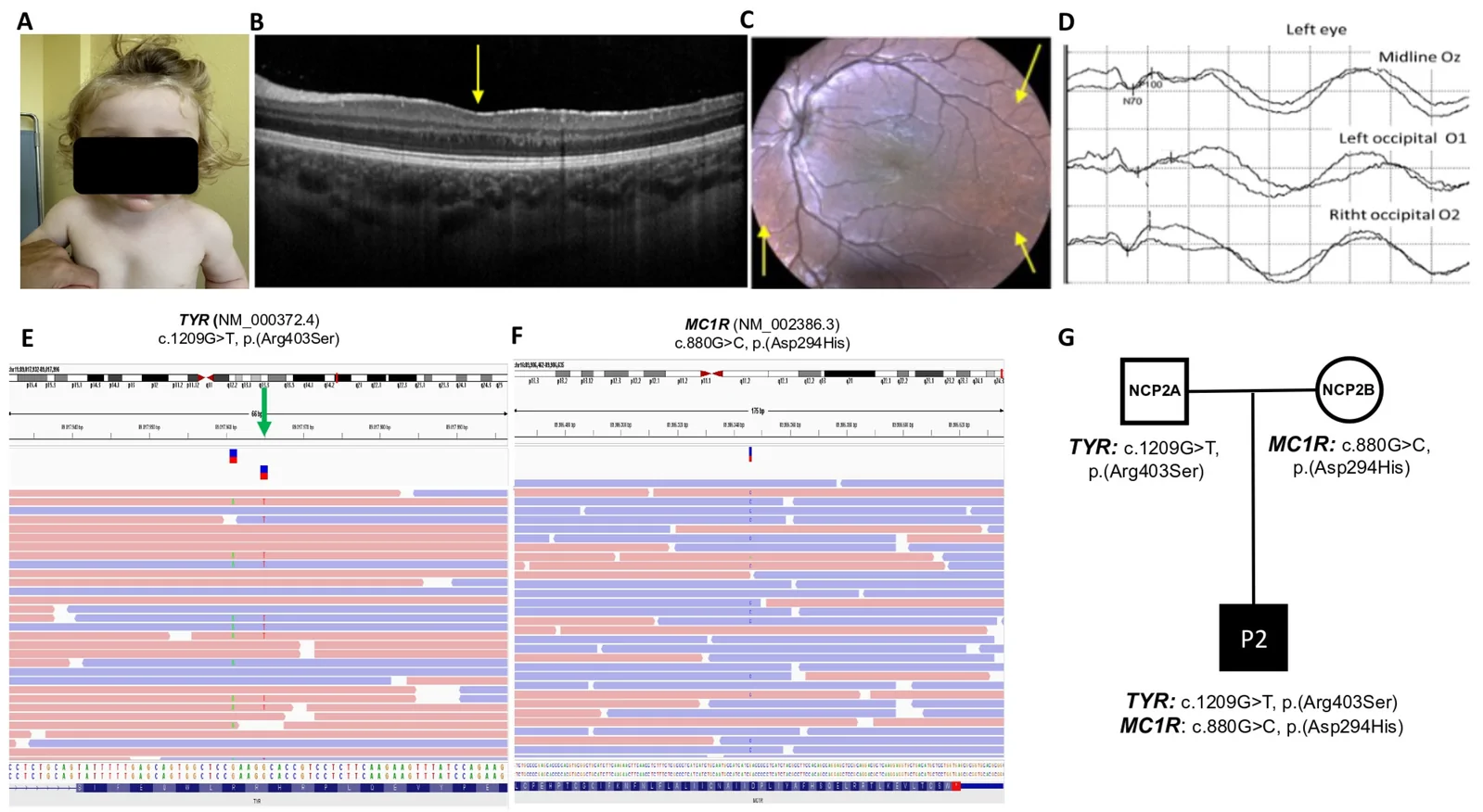
Clinical, genetic, and ophthalmological characterization of Proband P2. (**A**) Phenotypic features of the 3-year-old male proband (P2) showing a light complexion and dark-blond hair, without overt cutaneous signs of albinism. (**B**) Optical coherence tomography cross-section detailing a mild-to-moderate foveal hypoplasia, characterized by a very shallow but still present foveal pit (yellow arrow), persistence of inner retinal layers, and elongation of the outer segments. (**C**) Fundus photography demonstrating mild fundus hypopigmentation (grade 0–1) with partial visualization of the choroidal vessels only in some mid-peripheral retinal sectors (yellow arrows). (**D**) Flash visual evoked potentials, recorded at Oz, O1 and O2 occipital leads, showing the predominance of O2 activation compared to O1, consistent with excess of crossing fibers over the chiasm indicative of visual pathway misrouting. (**E**,**F**) Next-generation sequencing (NGS) data visualization (IGV) highlighting the digenic inheritance pattern: (**E**) the heterozygous missense variant c.1209G>T (p.Arg403Ser) in the *TYR* gene and (**F**) the heterozygous missense variant c.880G>C (p.Asp294His) in the *MC1R* gene—green arrows indicate the specific genomic coordinates of the variants. (**G**) Restricted family pedigree and data from trio segregation analysis confirming a candidate digenic genotype model with the paternally inherited *TYR*-c.1209G>T (NCP2A) and the maternally inherited *MC1R*-c.880G>C (NCP2B). Abbreviations: *TYR*: tyrosine.

**Figure 3:**
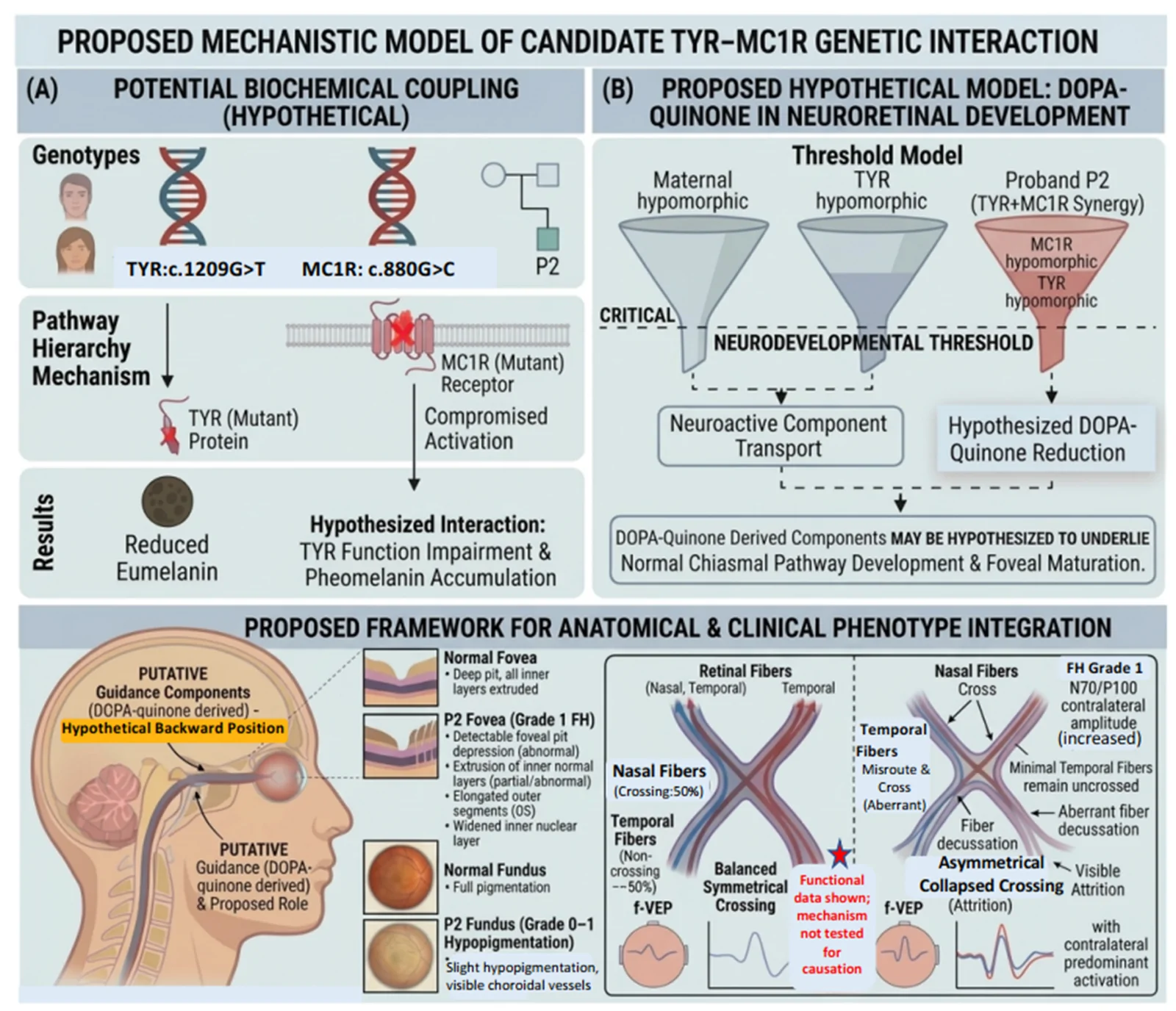
Proposed mechanistic model of candidate TYR–MC1R interaction in neuroretinal development (Proband P2). (*Note: This conceptual representation depicts a hypothesized model and has not been experimentally validated* in vitro *or* in vivo).

A 3-year-old boy (proband P2) was referred for genetic counseling following a series of routine pediatric ophthalmological evaluations scheduled for infantile nystagmus and impaired visual fixation (Figure 2). Strikingly, the extreme cutaneous or hair alterations typical of classic OCA were absent; however, unlike his parents who presented a darker phototype, the child exhibited a distinctly lighter complexion and dark-blond hair but without any evident reddish hue (Figure 2A, Table 1). The patient had been clinically monitored since the first year of life, leading to a definitive diagnosis of bilateral foveal hypoplasia (FH), associated with secondary FH-related nystagmus and reduced distance visual acuity. According to optical coherence tomography (OCT) classification standards, grade 1 of FH was anatomically characterized by an abnormal but still detectable foveal pit depression, associated with the extrusion of the inner retinal layers, elongation of the outer segment (OS) layer, and widening of the outer nuclear layer (ONL) (Figure 2B; Table 1) [43]. P2 proband displayed only mild and idiosyncratic signs of OA, namely, a slight fundus hypopigmentation (grade 0–1) with partial visualization of the choroidal vessels exclusively noticeable in some mid-peripheral retinal sectors [26] (Figure 2C; Table 1); an atypical chiasmal visual pathway misrouting pattern where, using flash visual evoked potentials (f-VEP), eye stimulation gave predominant activation (increased amplitude of the N70/P100 complex) over the contralateral occipital lead, consistent with prevalence of chiasmal crossed fibers over the uncrossed (Figure. 2D; Table 1) [29,30,31,32,33]. The custom multigene panel excluded causative biallelic sequential mutations in the classical albinism loci (*TYR*, *OCA2*, *GPR143*). Furthermore, tertiary bioinformatic analysis performed via the Emedgene platform focusing on copy number variations (CNVs) excluded the presence of occult heterozygous single-exon or multi-exon deletions or duplications on the second allele of the *TYR* gene, confirming the true monogenic heterozygous nature of the *TYR* locus. Instead, the analysis revealed a potential candidate model of digenic interaction involving heterozygous variants in two distinct pigmentation genes. Proband P2 was found to carry the known *TYR* missense variant c.1209G>T (p.Arg403Ser), directly implicated in the pathogenesis of non-syndromic OCA type 1 [44,45,46] (Figure. 2E), in combination with the known *MC1R* missense variant c.880G>C (p.Asp294His) (Figure 2F). While the latter is frequently identified in oncological cohorts presenting specific yellow/red pigmentary traits [1,2,3,4,5,6,7,8,9,10], it has never been previously reported in association with OCA/OA phenotypes [35,36,37,39]. No other pathogenic variants were identified in the *TYR* and *MC1R* genes, nor in any other genes included in the panel. Family-based trio segregation analysis confirmed a digenic inheritance pattern (Figure. 2G), demonstrating that the c.1209G>T variant in *TYR* was inherited from the father (NCP2A), while the c.880G>C variant in *MC1R* was inherited from the mother (NCP2B) (Table 1). Both non-consanguineous parents of P2 exhibited a darker hair phenotype (light brown) compared to their son and reported no personal history of ophthalmological or dermatological diseases. A proposed, hypothetical biochemical model for this potential interaction is schematically illustrated in Figure 3, depicting how partial reductions in both pathways might hypothetically lower early neuroactive intermediates (such as DOPA-quinone) below a critical developmental threshold required for normal chiasmal axon guidance and foveal maturation.

**Panel (A) Physiological Regulation & Biochemical Coupling (Hypothetical):** Schematic representation of candidate multigenic inheritance in proband P2. The combination of hypomorphic variants in *TYR* (c.1209G>T) and *MC1R* (c.880G>C) is hypothesized to lead to partial reductions in both receptor signaling and enzymatic output.

**Panel (B) Proposed Neurodevelopmental Threshold Model:** Visual representation of a hypothesized neurodevelopmental threshold. While single heterozygous variants may maintain pathway activity above a developmental threshold, a combined partial reduction in both *TYR* and *MC1R* pathways is hypothesized to reduce DOPA-quinone-derived neuroactive components below this threshold, potentially impacting chiasmal axon guidance and foveal maturation.

Proposed Anatomical & Clinical Phenotype Integration (Bottom Panel):

- **Anatomical Localization (Left):** Sagittal representation of the visual pathway showing the optic nerve extending toward the optic chiasm, where melanogenesis-derived signals are hypothesized to regulate axonal guidance.
- **Foveal & Fundus Phenotype (Center):** Comparison between a normal deep foveal pit and the patient’s Grade-1 Foveal Hypoplasia (FH), alongside fundus hypopigmentation (Grade 0–1).
- Atypical Chiasmal Visual Pathway Misrouting Pattern (Right):

1. Normal Chiasm: Shows a balanced, symmetrical crossing where nasal fibers cross (≈50%) and temporal fibers remain uncrossed (≈50%), resulting in symmetrical bilateral cortical activation under flash visual evoked potentials (f-VEP).
2. P2 Candidate Interaction Chiasm: Illustrates the proposed atypical misrouting pattern where temporal fibers aberrantly decussate (cross) due to hypothesized insufficient guidance components. This structural asymmetry leads to a reduction in uncrossed fibers (visible attrition) and a predominant contralateral cortical activation (graphically shown by the amplitude increase in the contralateral N70/P100 f-VEP wave) [29,30,31,32,33].

Abbreviations: *TYR*: tyrosinase; *MC1R*: melanocortin 1 receptor; MITF: microphthalmia-associated transcription factor; DOPA: dihydroxyphenylalanine; OCT: optical coherence tomography; f-VEP: flash visual evoked potentials.

## 3. Discussion

OCA and OA comprise a heterogeneous group of hypopigmentation disorders characterized by impaired melanin synthesis within the skin, hair, and eyes [21]. Given the significant clinical overlap among distinct subtypes, molecular genetic testing via targeted NGS has become essential for accurate diagnosis and counseling. In the present study, within a screening cohort of 48 Caucasian individuals evaluated for hypopigmentation and ocular features, we characterized two selected probands carrying compound or digenic *MC1R*-associated genotypes presenting strikingly different pathological manifestations. Both cases shared the hypomorphic c.880G>C (p.Asp294His) allele, a known red hair color (RHC) variant that shifts melanin synthesis toward pheomelanogenesis [1,2,3,10]. However, the clinical expressivity observed in these individuals was strongly modulated by their respective multigenic architectures. In proband P1, genetic testing identified compound heterozygous *MC1R* variants, where the maternal p.Asp294His allele was paired with a novel, paternally inherited frameshift mutation (c.537dupC, p.Ile180Hisfs*59) predicted to induce a loss of receptor function. Proband P1 exhibited a classic pheomelanic phototype (bright red hair, fair skin with freckles, and light irises) in the absence of pathognomonic ophthalmic signs of albinism. Notably, the patient had a history of an early-onset GBCC surgically removed at 30 years of age. While *MC1R* variants are well-established independent risk factors for NMSC [11,12,13], the occurrence of GBCC in a single compound heterozygous individual cannot, by itself, establish a direct causal relationship between this genotype and GBCC development. Family segregation confirmed that P1 carries *MC1R* variants inherited in *trans*. Furthermore, while the personal histories of late-onset cutaneous melanoma reported in both heterozygous parents are compatible with the known association between *MC1R* hypomorphic alleles and melanoma risk [6,7,8,11], they represent clinical consistency rather than formal cosegregation evidence for the proband’s specific tumor histotype. Conversely, proband P2 presented an atypical, sight-threatening ophthalmological phenotype that raises the possibility of a novel candidate digenic mechanism. Evaluated at early pediatric age for infantile nystagmus and impaired visual fixation, the child exhibited bilateral horizontal pendular nystagmus, high hyperopia, and reduced distance visual acuity. Macular OCT morphologically detailed grade-1 FH in both eyes. Electrophysiological assessments of the visual pathway via multichannel monocular f-VEPs documented an atypical distribution of cortical responses over occipital leads, suggesting a potential pattern of chiasmal visual pathway misrouting [29,30,31,32,33]. Targeted NGS excluded biallelic defects in classical albinism loci (*TYR*, *OCA2*, *GPR143*), as well as CNVs across a targeted retinal gene panel. Instead, molecular investigation identified the coexistence of a single, paternally inherited heterozygous *TYR* hypomorphic variant (c.1209G>T, p.Arg403Ser) [47,48,49,50] and the maternally inherited *MC1R* p.Asp294His allele. Biochemically, the MC1R signaling pathway directly drives the downstream transcriptional activation of *TYR*, tyrosinase-related protein 1 (*TYRP1*), and dopachrome tautomerase (*DCT*) via the MITF axis [51]. The prior literature established that *MC1R* variants alone are insufficient to cause reduced visual function or macular abnormalities in red-haired individuals, indicating that MC1R pathways do not independently direct neuroretinal development [15,33]. However, as previously inferred in cohorts screening *GPR143*, *TYR*, and *OCA2* mutations, alterations in primary albinism genes may affect visual pathway development either directly or through interactions with genetic modifiers like *MC1R* [35]. Specifically, current neurobiological models hypothesize that early upstream products of the melanin pathway—up to DOPA-quinone—or the precise distribution of their derived neuroactive components contribute to optic chiasm axonal guidance and foveal maturation [15,32,35]. In proband P2, we hypothesize that a concurrent, partial reduction in both the structural enzyme (*TYR*) and its upstream membrane receptor (*MC1R*) could theoretically lower early melanogenesis products below a critical functional threshold required for typical visual pathway development (Figure 3). This hypothesized metabolic synergy is also clinically reflected in the child’s pigmentary phenotype: unlike his parents, who presented a darker, light-brown hair color without ocular issues, proband P2 exhibited a lighter phototype (fair skin and dark-blond hair with a golden hue).

To date, the role of MC1R as a genetic modifier in albinism spectrum disorders has been reported primarily in combination with heterozygous mutations in the *OCA2* gene [15,35]. Our findings expand this conceptual framework by proposing that *MC1R* could potentially act as a phenotypic modifier in patients carrying a single candidate *TYR* variant. Some inherent methodological considerations of this work should be noted. As a descriptive report focused on two detailed family pedigrees, the proposed candidate *TYR*–*MC1R* interaction warrants further evaluation in larger, independent patient cohorts. Additionally, while targeted NGS panel sequencing excluded known structural and coding variants across major albinism genes, complex non-coding or deep-intronic regulatory variations elsewhere in the genome cannot be completely ruled out. In the absence of direct in vitro or in vivo functional assays evaluating signaling kinetics or enzymatic yield, the hypothesized quantitative threshold model for neuroretinal development remains an interpretive, theoretical framework. Furthermore, electrophysiological findings in pediatric patients based on f-VEP require cautious interpretation regarding chiasmal misrouting compared to pattern-VEP or histological approaches [29,30,31]. In conclusion, our study highlights the potential role of oligogenic mechanisms in clarifying complex or atypical cases of congenital ocular disorders. Incorporating *MC1R* and other pigmentation modifier genes into routine diagnostic NGS panels may help refine genotype–phenotype correlations and prevent hypomorphic or oligogenic forms of foveal hypoplasia from being molecularly overlooked. Furthermore, given that *MC1R* variants impair photoprotection, identifying these genetic modifiers has practical clinical relevance by prompting lifelong dermatological surveillance for skin cancers. However, further large-scale multi-center studies and functional experiments remain necessary to confirm whether *TYR*–*MC1R* combinations represent a true causative digenic mechanism or a subtle phenotypic modifier effect.

## 4. Methods

### 4.1. Ethics Approval and Consent to Participate

According to the Italian Medicines Agency (AIFA) Determination No. 425 of 8 August 2024, “Guidelines for the classification and conduct of observational studies on medicinal products” (Official Journal of the Italian Republic, General Series No. 194, 20 August 2024), the present work does not qualify as an observational study, as it consists of data collection/analysis without a clinical study methodological design. Therefore, the regulation does not apply and Ethics Committee approval was not required. Personal health data were processed in compliance with the Code of Conduct for the use of health data for educational and scientific publication purposes, adopted by the Veneto Region (Regional Government Resolution No. 1633/2022). All data were anonymized prior to analysis. This study was conducted in accordance with the tenets of the Declaration of Helsinki.

### 4.2. Patient Recruitment and Clinical Workup

The probands and their respective first-degree family members were recruited and clinically evaluated at the Camposampiero Hospital (Azienda ULSS 6 Euganea, Padova, Italy). Each patient underwent a comprehensive dermatologic examination, including accurate anamnestic familial and medical history retrospective retrieval focused on several cutaneous conditions, such as symptoms, abnormal events and/or ascertained diseases. Moreover, complete ophthalmic and orthoptic examinations were performed to particularly assess: ocular anamnesis; axial refraction and equivalent spherical diopter (ESD) using automated tool and cycloplegic retinoscopy; best-corrected visual acuity (BCVA) measured in Snellen equivalents by standardized, age-appropriate, logarithmic charts at a test distance of three meters; eye anterior segment and structural evaluation of iris transillumination defects using slit-lamp biomicroscopy; fundus oculi after pupil dilatation with evaluation of retinal hypopigmentation by means of fundus photography with confocal device (EIDON, Centervue, Padova, Italy); foveal architecture via optical coherence tomography of the macula (Spectralis HRA, Heidelberg Engineering, Heidelberg, Germany). Whether one or more of these non-invasive exams documented pathological patterns, the functional status of both retina and optic visual pathways was respectively evaluated using full-field electroretinography and flash visual evoked potentials. These electrophysiologic examinations were carried out and recorded by the Retimax Plus system (Costruzioni Strumenti Oftalmici, Florence, Italy) according to the standards of the International Society for Clinical Electrophysiology of Vision (ISCEV) [52,53]. 

### 4.3. DNA Extraction and NGS High-Throughput Screening

Peripheral blood samples were collected from both probands and their respective parents. Genomic DNA (gDNA) was isolated from peripheral blood leukocytes using automated magnetic-bead technology according to the manufacturer’s standard protocols. gDNA concentration, purity, and structural integrity were quantitatively assessed using a Qubit Fluorometer (Thermo Fisher Scientific, Waltham, MA, USA) and spectrophotometric analysis. Targeted enrichment and library preparation were performed using Agilent SureSelect technology (Agilent Technologies, Santa Clara, CA, USA) following the manufacturer’s instructions. High-throughput sequencing was executed on the NextSeq 2000 platform (Illumina, San Diego, CA, USA) utilizing paired-end reads to guarantee optimal target coverage depth and analytical precision. The targeted sequencing architecture was divided into two comprehensive, clinical-grade screening tiers tailored to distinct pathophysiological manifestations: i. Nonsyndromic Oculocutaneous Albinism and Ocular Albinism Panel comprising 23 specific core pigmentary loci directly involved in melanin biosynthesis and melanosome trafficking (including *TYR*, *OCA2*, *GPR143*, and *MC1R*), and ii. Inherited Retinal Dystrophies and Ocular Development Panel comprising an expanded sub-panel of 312 genes known to overlap with congenital visual disorders, foveal hypoplasia, and developmental maculopathies. 

### 4.4. Bioinformatics Pipeline, Variant Filtering, and In Silico Functional Annotation

Raw sequencing data underwent secondary analysis using the Illumina DRAGEN Bio-IT Platform (v4.3; Illumina), which performed high-efficiency adapter trimming, low-quality base filtering, and duplicate removal. High-quality reads were aligned to the human reference genome assembly GRCh38 (hg38). Comprehensive quality metrics, including mean depth of coverage, percentage of target bases covered at ≥30×, and coverage uniformity across target amplicons and gene loci, were verified via the diagnostic pipeline. Tertiary analysis, variant annotation, and clinical interpretation were managed using the Emedgene platform (v37.0.2; Illumina). To systematically rule out the presence of missing heritability caused by genomic structural rearrangements on single-coding alleles, CNVs, including single-exon and multi-exon deletions or duplications, were computationally evaluated across all target genes. This was performed using validated depth-of-coverage analysis algorithms embedded within the Emedgene and DRAGEN pipelines, which compare normalized read depths against a baseline cohort. The analytical pipeline achieved a high-resolution window capable of identifying structural variations down to the single-exon level. Structural and functional impacts of candidate variants were predicted in silico utilizing multiple algorithmic predictors (including PolyPhen-2, SIFT, MutationTaster, and CADD scores) and interconnected genomic databases (such as gnomAD, ClinVar, dbSNP, and the Human Gene Mutation Database). For novel frameshift alterations, truncating effects and premature termination codons were verified. Candidate variants were finally classified according to the joint consensus guidelines recommended by the American College of Medical Genetics and Genomics and the Association for Molecular Pathology (ACMG/AMP) [41].

### 4.5. Familial Segregation Analysis

To determine the phase of the identified candidate mutations and establish the monogenic compound heterozygosity or digenic multi-locus inheritance models, family-based trio segregation analysis was conducted. Candidate variants identified in proband P1 and proband P2 were analyzed in gDNA samples extracted from their respective non-consanguineous parents using targeted bidirectional Sanger sequencing or direct NGS segregation mapping, validating the maternal and paternal inheritance patterns.

## Figures and Tables

**Table 1:** Clinical, refractive, and molecular findings of the investigated cohorts and their family members.

ID/Sex/Age	Pathologic Gene Variants	Pathologic Phenotype/Age at Onset (Y)	Pigment Hair	Iris Color	Refraction (ESD)		BCVA (SE)	Nystagmus	IT/ F. Hypo.	FH	fVEP	ff-ERG-Alteration	Previous Phenotype-Related Reports
					OD OS	OD OS	OD OS						
P1/M/50 Y	*MC1R* c.537dupC *MC1R* c.880G>C	GBCC (NMSC)/30 Y	Red	Blue-green	−4.75 * −4.75 *	20/20 20/20	20/50 § 20/63 §	No	No	No	NR	NR	Refs: [12,13]
NCP1A/M/82 Y	*MC1R* c.880G>C	MSC/73 Y	Dark blond	Blue	NR NR	NR NR	NR NR	NR	NR	NR	NR	NR	None
NCP1B/F/78 Y	*MC1R* c.880G>C	MSC/76 Y	Light brown	Light brown	NR NR	NR NR	NR NR	NR	NR	NR	NR	NR	Refs: [6,7,8]
P2/M/3 Y	*TYR* c.1209G>T *MC1R* c.880G>C	FH and CN/1 Y	Dark blond, golden-reddish	Light brown	+9.75 +9.75	20/50 § 20/63 §	Yes Yes	No	No	Yes	Yes	No	Refs: [42,43,44]
NCP2A/M/31 Y	*TYR* c.1209G>T	None/NA	Light brown	Light brown	NR NR	NR NR	NR NR	NR	NR	NR	NR	NR	NA
NCP2A/F/29 Y	*MC1R* c.880G>C	None/NA	Brown	Blue	NR NR	NR NR	NR NR	NR	NR	NR	NR	NR	NA

ID, identification code of probands or their parents; P, proband; NCP, non-consanguineous parent; Y, years; M, male; F, female; *MC1R*, melanocortin-1 receptor gene; *TYR*, tyrosinase gene; GBCC, giant basal cell carcinoma; NMSC, non-melanoma skin cancer; MSC, melanoma skin cancer; FH, foveal hypoplasia; CN, congenital nystagmus; NA, not applicable; ESD, equivalent spherical diopters; OD, oculus dexter (right eye); OS, oculus sinister (left eye); *, ESD prior to corneal refractive surgery; NR, not recorded; BCVA, best-corrected visual acuity; SE, Snellen equivalent; §, BCVA measured by tumbling E test after an accurate learning assessment; f-VEP, flash visual evoked potentials; ff-ERG, full-field electroretinography.

## Data Availability

All data generated or analyzed during this clinical study are included in this article. The datasets generated and analysed during the current study are available in the ClinVar repository and Database of Genomic Variants, i.e.: https://www.ncbi.nlm.nih.gov/clinvar/variation/357699/; accessed on the 28 July 2026. https://dgv.tcag.ca/dgv/app/variant?id=nsv603391&ref=GRCh37/hg19: accessed on the 28 July 2026. All authors agree to make the materials, data and associated procedures promptly available to readers. Further enquiries can be directed to the corresponding author.
